# A Rare Case of HIV-Associated Plasmablastic Lymphoma of Anal Canal

**DOI:** 10.7759/cureus.17782

**Published:** 2021-09-06

**Authors:** Muhamed Tajudeen, Souradeep Dutta, Ankit Jain, Bheemanathi Hanuman Srinivas, Vishnu Prasad Nelamangala Ramakrishnaiah

**Affiliations:** 1 Surgery, Jawaharlal Institute of Postgraduate Medical Education and Research, Puducherry, IND; 2 Pathology, Jawaharlal Institute of Postgraduate Medical Education and Research, Puducherry, IND

**Keywords:** anal growth, aids related lymphoma, lymphoma of anal canal, extraoral plasmablastic lymphoma, plasmablastic lymphoma

## Abstract

Plasmablastic lymphoma, an acquired immunodeficiency syndrome defining malignancy, is a subtype of diffuse large B-cell lymphoma. It is classically described in the oral cavity, the extraoral presentation being rare. Owing to its rarity and aggressiveness, plasmablastic lymphoma poses a diagnostic and therapeutic challenge to the treating physician. A 40-year-old male, recently diagnosed with HIV infection, presented with bleeding per rectum and spurious diarrhea. Examination revealed proliferative growth in the anal canal. Biopsy of the lesion was diagnostic of plasmablastic lymphoma. Computed tomography and magnetic resonance imaging were done to stage the lesion and assess the local extent, respectively. A multidisciplinary board discussion was done, and the patient was instituted on antiretroviral therapy and chemoradiotherapy. Following six cycles of chemotherapy and 25 fractions of radiotherapy, he achieved complete remission.

## Introduction

Plasmablastic lymphoma (PBL), classified as a subtype of diffuse large B-cell lymphoma (DLBCL) by the World Health Organization (WHO), is a rare and aggressive malignancy, commonly seen in patients with human immunodeficiency virus (HIV) infection [1]. It is classically described in the oral cavity, the extraoral presentation being rare. Owing to its rarity and aggressiveness, PBL poses a diagnostic and therapeutic challenge to the treating physician. Here, we discuss a case of PBL of the anal canal, an exceedingly rare variant with an outline of the literature.

## Case presentation

**Figure 1:**
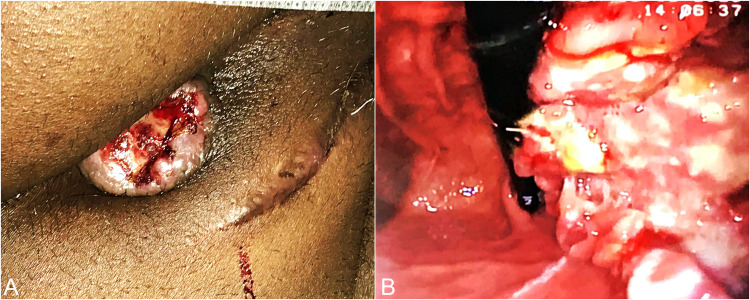
Clinical photographs (A) Polypoidal growth seen protruding from the anal canal. (B) Colonoscopy showing a view of the anal canal growth.

A 40-year-old male, known case of HIV, presented to the surgical emergency with complaints of bleeding per rectum for the past 15 days, containing both fresh blood and clots, associated with the painful passage of stools and spurious diarrhea. On examination, he was pale and moderately built. Vitals were normal. Per abdominal examination revealed no significant findings. Per rectal examination revealed a polypoidal growth from 1 o'clock to 3 o'clock starting from the anal verge, upper margin not palpable (Figure 1A). There was no palpable inguinal or generalized lymphadenopathy. Blood investigation revealed anemia (hemoglobin 9.2 g/dl) and normal liver and renal function tests. Carcinoembryonic antigen level was not elevated (0.8 ng/ml). The CD4 count was 110 cells/mm^3^.

After the initial investigations, a colonoscopy was done which showed a growth in the distal rectum and anal canal for a length of 5 cm from 1 o'clock to 3 o'clock position (Figure 1B). Rest of the colon was normal. 

**Figure 2:**
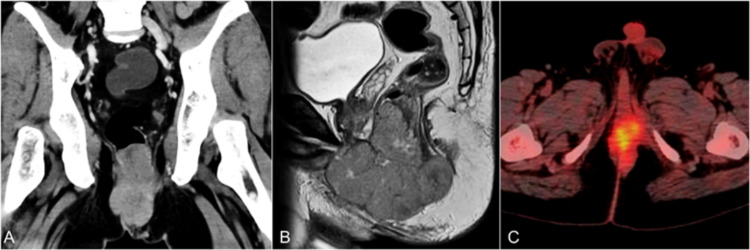
Pretreatment cross-sectional imaging (A) Coronal reconstruction of CECT showing a heterogeneously enhancing soft-tissue density lesion measuring 9.1 x 5.4 x 7.8 cm involving the anal canal with loss of fat plane with levator ani bilaterally. (B) Sagittal reconstruction of T2 MRI showing a large lobulated lesion involving the anal canal with extension into the perineum and anorectal junction. (C) FDG-PET CT fusion image showing metabolically active disease in the anal canal. CECT: contrast-enhanced computed tomography, MRI: magnetic resonance imaging, FDG-PET: fluorodeoxyglucose positron emission tomography.

Contrast-enhanced computed tomography (CECT) showed a heterogeneously enhancing soft-tissue density lesion measuring 9.1 x 5.4 x 7.8 cm involving the anal canal with loss of fat plane with levator ani bilaterally, extending till the root of the penis and posterior urethra anteriorly, puborectalis posteriorly, anorectal junction superiorly, and inferiorly till the subcutaneous tissue in the perianal region (Figure 2A). Several enlarged lymph nodes were noted in inguinal, iliac, and retroperitoneal lymph node basins. Magnetic resonance imaging (MRI) was done, which showed a large lobulated lesion involving the anal canal with extension into the perineum and anorectal junction measuring 8.7 x 5.5 x 8.8 cm (Figure 2B). Fluorodeoxyglucose positron emission tomography (FDG-PET) was done suggestive of metabolically active disease in the anal canal and perianal subcutaneous tissue with no uptake in inguinal, iliac, and retroperitoneal lymph node basins or bone marrow (Figure 2C).

**Figure 3:**
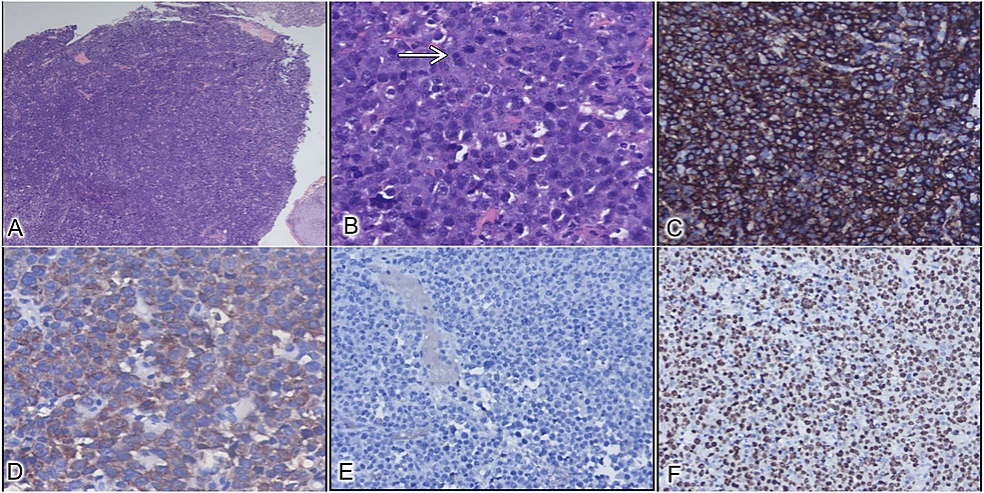
Histopathological images (A) Sheets of tumor cells along with overlying normal squamous epithelium (H&E X40). (B) Tumor cells are polygonal in shape with an eccentrically placed nucleus and frequent mitotic figures (white arrow) (H&E X400). (C) Immunohistochemical staining shows strong membranous CD138 positivity (DAB X100). (D) Tumor cells positive for lambda light chain (DAB X200). (E) Tumor cells are negative for pan-cytokeratin, LCA, CD20, CD30, and Kappa light chain (H&E X100). (F) Tumor cells showing high Ki67, 90% (DAB X100). H&E: hematoxylin and eosin stain, DAB: 3,3′-diaminobenzidine immunohistochemical stain, LCA: leucocyte common antigen, CD: cluster of differentiation.

Biopsy from the lesion showed a monomorphic population of large round cells with areas of tumor necrosis and hemorrhage (Figure 3A-3F).

The cells were positive for CD38 and CD138 and negative for CD20 and CD79a. Based on morphological and immunohistochemistry (IHC) features, the biopsy was consistent with PBL. The Ki67 score was >90%.

**Figure 4:**
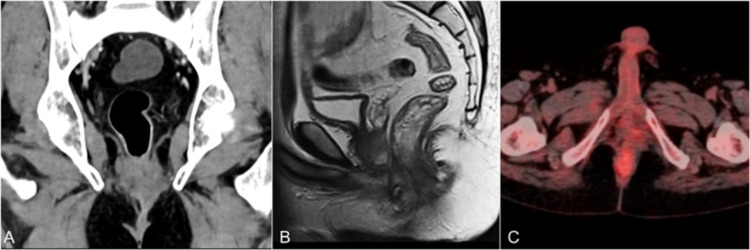
Posttreatment cross-sectional imaging (A) CT, (B) MRI, and (C) PET images showing resolution of the tumor. CT: computed tomography, MRI: magnetic resonance imaging, PET: positron emission tomography.

The patient was started on highly active antiretroviral therapy (HAART) after discussion with the antiretroviral therapy clinic. After a multidisciplinary tumor board discussion, he was planned for a dose-adjusted etoposide, prednisone, vincristine, cyclophosphamide, doxorubicin (DA-EPOCH) regimen followed by radiotherapy. He received six cycles of DA-EPOCH chemotherapy with granulocyte colony-stimulating factor (G-CSF) support followed by 45Gy radiotherapy over 25 fractions. Following chemoradiation, he was assessed for response. Sigmoidoscopy showed indurated area at the previous tumor site with no residual mass; multiple biopsies were reported to be fibrotic tissue with no evidence of tumor. Repeat cross-sectional imaging (CECT, MRI, and FDG-PET) after the chemoradiotherapy also showed clinically complete response (Figure 4A-4C). He is currently under a six-month follow-up on HAART. 

## Discussion

Acquired immunodeficiency syndrome (AIDS) is known for an increased incidence of lymphoproliferative disorders due to the interplay of HIV-associated poor immune response and infection by oncologic viruses [1]. Non-Hodgkin’s lymphomas are frequently described with AIDS. DLBCL is the most common B-cell lymphoma and accounts for 41% of non-Hodgkin’s lymphomas [1]. PBL is a subtype of DLBCL that accounts for 2.6% of AIDS-related lymphomas [2]. The causative agent of PBL is the Epstein-Barr virus (EBV); however, its association with human herpesvirus 8 (HHV-8) is still an enigma [2].

The usual presentation of PBL is around 40 years of age with male preponderance (4:1) [3]. However, few pediatric cases have been reported as well [4]. PBL has been commonly described within the oral cavity, rarely in the extraoral sites such as the nose and paranasal sinuses, gastrointestinal tract, skin and soft tissue, and lymph nodes [3]. Although the incidence is remarkably higher in the people living with HIV/AIDS (PLHA) population, PBL has also been described in immunocompetent individuals [5]. HIV-associated PBL is usually seen with individuals whose CD4 count is around 200 cells/mm^3^, mean duration of the disease is eight years, and average viral load is over 2,50,000 copies/ml [6]. PBL of the anal canal is usually a rapidly growing tumor with the presentation in an advanced clinical stage. The patients usually present with bleeding per rectum, tenesmus, and change in bowel habits [7]. Moreover, "B symptoms" are reported in almost one-third to half of these patients.

The staging system used for PBL is the Ann-Arbor classification, and most of the patients belonged to either stage 1 or stage 4 disease [1]. The initial and most widely used staging investigation for lymphomas is CECT of the neck, thorax, and abdomen. However, MRI is the investigation of choice for anal canal lymphomas due to its superior diagnostic ability for invasion of surrounding structures. The lesion usually shows poor contrast uptake and diffusion restriction, showing homogeneous intermediate T1 signal and an intermediate/high T2 signal [8,9]. Moreover, MRI can also detect bone marrow involvement, seen in one-third of patients with PBL [10]. Bone marrow involvement upstages the disease and is associated with poor prognosis [11]. Although a CECT of the neck, thorax, and abdomen also helps assess response to treatment, FDG-PET remains the mainstay in assessing response to chemotherapy in FDG-avid lymphomas such as PBL, as per the Lugano 2014 criteria [12,13]. However, an FDG-PET might not always be reliable in HIV-associated lymphomas due to associated infection and inflammatory process. Therefore, an additional CECT scan can be done in such cases.

Histopathology remains the gold standard in the diagnosis of PBL. Under microscopy, they show a monomorphic proliferation of plasmacytoid cells (eccentric nucleus, abundant cytoplasm, and prominent central nucleolus) in a starry sky background which again reiterates the aggressive nature of these tumors [2]. The key pathologic feature is the absence of B-cell markers (CD4 and CD20) and the presence of plasma cell markers (CD38 and CD138). EBV-encoded RNA (EBER) has been used for its diagnostic association [2]. c-MYC rearrangements often described in high-grade lymphomas are also noted with PBL [2]. The proliferative index (Ki67 > 60%) is usually high, correlating with aggressive nature.

In cases of HIV-positive PBL, HAART initiation has been shown to have better outcomes. Cyclophosphamide, doxorubicin, vincristine, and prednisone (CHOP) regimen has been tried extensively in the past. However, it has been abandoned due to poor survival outcomes in HIV-associated PBL [1]. Given its superior remission rates, DA-EPOCH is the preferred chemotherapeutic regimen for PBL. Although individual reports have described radiation therapy; however, there is no proven additional benefit [1,14]. Patients with good responses to HAART can be given EBV-targeted therapies [15]. Bortezomib proteasome inhibitor might have a positive outcome and is still under evaluation [16]. The results on stem cell transplantation are promising in achieving remission following the chemotherapy [1].

## Conclusions

Although rare, PBL should be a differential diagnosis for immunocompromised patients presenting with a suspicious neoplastic lesion of the anal canal. Given the aggressive biological behavior of the tumor, the aim is to have an early diagnosis of the condition. Timely initiation of appropriate therapy can have good treatment results.
